# Cost-effectiveness model comparing olanzapine and other oral atypical antipsychotics in the treatment of schizophrenia in the United States

**DOI:** 10.1186/1478-7547-7-4

**Published:** 2009-04-07

**Authors:** Nicolas M Furiak, Haya Ascher-Svanum, Robert W Klein, Lee J Smolen, Anthony H Lawson, Robert R Conley, Steven D Culler

**Affiliations:** 1 Medical Decision Modeling Inc., Indianapolis, IN, USA; 2 Eli Lilly and Company, Indianapolis, IN, USA; 3 Lilly USA, LLC, Indianapolis, IN, USA; 4 Emory University, Atlanta, GA, USA

## Abstract

**Background:**

Schizophrenia is often a persistent and costly illness that requires continued treatment with antipsychotics. Differences among antipsychotics on efficacy, safety, tolerability, adherence, and cost have cost-effectiveness implications for treating schizophrenia. This study compares the cost-effectiveness of oral olanzapine, oral risperidone (at generic cost, primary comparator), quetiapine, ziprasidone, and aripiprazole in the treatment of patients with schizophrenia from the perspective of third-party payers in the U.S. health care system.

**Methods:**

A 1-year microsimulation economic decision model, with quarterly cycles, was developed to simulate the dynamic nature of usual care of schizophrenia patients who switch, continue, discontinue, and restart their medications. The model captures clinical and cost parameters including adherence levels, relapse with and without hospitalization, quality-adjusted life years (QALYs), treatment discontinuation by reason, treatment-emergent adverse events, suicide, health care resource utilization, and direct medical care costs. Published medical literature and a clinical expert panel were used to develop baseline model assumptions. Key model outcomes included mean annual total direct cost per treatment, cost per stable patient, and incremental cost-effectiveness values per QALY gained.

**Results:**

The results of the microsimulation model indicated that olanzapine had the lowest mean annual direct health care cost ($8,544) followed by generic risperidone ($9,080). In addition, olanzapine resulted in more QALYs than risperidone (0.733 vs. 0.719). The base case and multiple sensitivity analyses found olanzapine to be the dominant choice in terms of incremental cost-effectiveness per QALY gained.

**Conclusion:**

The utilization of olanzapine is predicted in this model to result in better clinical outcomes and lower total direct health care costs compared to generic risperidone, quetiapine, ziprasidone, and aripiprazole. Olanzapine may, therefore, be a cost-effective therapeutic option for patients with schizophrenia.

## Background

Schizophrenia is often a debilitating, persistent, and costly disorder. Although it afflicts only about 1% of the U.S. population [1], it imposes a disproportionately large economic burden relative to other mental illnesses and nonpsychiatric medical disorders [2]. The most recent cost-of-illness study in the United States [3] estimated schizophrenia to cost $62.7 billion in the year 2002, with total direct medical costs being driven primarily by the utilization of health care resources in treating symptom relapses.

Antipsychotics are considered the core treatment regimen for schizophrenia, aimed at reducing the risk of relapse and enhancing long-term functional outcomes [4]. Although patients are expected to be on their medications for a prolonged time – often a lifetime [4], a majority (58%) of patients are nonadherent to antipsychotic therapy [5]. Studies have shown that nonadherence to antipsychotic therapy is associated with an increased risk of relapse and inpatient psychiatric hospitalization [6-14], the costliest components in treating schizophrenia [15-19].

Studies examining adherence among patients with schizophrenia have demonstrated that adherence is not an "all or none" phenomenon because many patients appear to be partially adherent [7,20,21], not taking their medications as prescribed, and/or having gaps in medication intake [16,18,20,22]. Prior research [23-25] has documented the dynamic nature of treatment with antipsychotics where patients start, switch, continue, and discontinue their antipsychotics for various reasons, including patient decision, lack of medication efficacy, and medication intolerability.

A large number of studies have found different adherences [26-32] and persistence [23-25,33-51] among antipsychotic medications. Although it was long believed that patients with schizophrenia discontinue their medications primarily due to treatment-emergent adverse events, more recent studies have reported that lack of medication efficacy is a more prevalent driver of treatment discontinuation compared to medication intolerability [23-25,52]. Furthermore, patients who experience better treatment outcomes tend to perceive their medication as more beneficial and are more likely to persist taking them [53-55]. As a result, the differential clinical benefits among antipsychotic medications have a variety of cost-effectiveness implications for patients, third-party payers, and society.

Most prior research on the cost-effectiveness of antipsychotics in the treatment of schizophrenia has compared first-generation antipsychotics (FGAs) and second-generation antipsychotics (SGAs) [17,49,56,57]. Although studies have reached different conclusions regarding the cost-effectiveness of 1 or more SGAs versus FGAs [17,49,57], the debate about the relative benefits of FGAs versus SGAs has become less relevant for U.S. payers, who may have little incentive to use FGAs following patent expiry of risperidone and its availability in generic form and lower cost. The economic environment appears to be changing after oral risperidone, the most frequently used SGA for the treatment of schizophrenia in the United States, has become available in generic form in July 2008. We anticipate increased interest in cost-effectiveness models that compare generic oral risperidone with other frequently used oral SGAs to address payers' questions concerning the relative cost-effectiveness of the various SGAs given the growing economic constraints in the U.S. health care system.

The broad objective of this study is to create an economic decision model to compare the relative clinical benefits, associated direct medical costs, and cost-effectiveness of oral olanzapine, oral generic risperidone (primary comparator), quetiapine, ziprasidone, and aripiprazole in the usual treatment of schizophrenia from the perspective of third-party payers in the U.S. health care system.

In this paper, we first present a conceptual structure of the model and identify sensitivity analysis conducted. We then review baseline assumptions for key clinical and economic inputs. Next, we report results for the baseline assumptions and the results of 1-way sensitivity analyses where discrete changes in the input values for key variables are evaluated for their impact on results. We also include results of probabilistic sensitivity analyses (PSA) where inputs for multiple variables are sampled from distributions for multiple cohorts. The paper concludes with a discussion, limitation of the model, and summary.

## Methods

### Model Structure and Study Design

A Monte Carlo Microsimulation (MCM) model was developed to compare the cost-effectiveness of 5 frequently used oral atypical antipsychotics in the usual care of schizophrenia in the United States. Results are based upon a simulation of 1,000,000 patients. The target patient population was community-dwelling adult patients with schizophrenia who had a history of schizophrenia. The model compares oral olanzapine with generic oral risperidone (primary comparator), quetiapine, ziprasidone, and aripiprazole in the treatment of patients with schizophrenia for a 1-year study period. Health care costs are evaluated from the perspective of a public or private third party health care payer in the United States. The model simulates the dynamic nature of usual care where patients switch, continue, discontinue, and restart their antipsychotics in quarterly cycles. The choice of quarterly cycles is based on previous cost-effectiveness research [58] and expert consensus that the duration of an "adequate antipsychotic treatment trial" [25,58,59] is 3–8 weeks if there is no response and 5–12 weeks if there is a partial response before switching to another pharmacologic strategy. The MCM model captures clinical outcomes and estimates third-party payers' costs. The MCM model allows for a number of input parameters including: adherence levels, relapse with and without hospitalization, health state utilities, treatment discontinuation by reason, treatment-emergent adverse events, health care resource utilization, and health care costs, including medication costs. Key clinical outcomes predicted include psychiatric inpatient hospitalization rates and quality-adjusted life years (QALYs). Costs are expressed in U.S. dollars based on 2007 values. The MCM model assumes an intent-to-treat approach that attributes all estimated direct medical costs to the initial therapy.

Although schizophrenia is a chronic illness that requires long-term treatment, we chose a 1-year timeframe for the MCM model because 1 year is the time period the typical third-party payer is responsible for covering medical costs of a covered life. In addition, the dynamic nature of the treatment for schizophrenia with its high rate of medication switching and discontinuation makes it difficult to directly relate the initial treatment selection to the final cost-effectiveness outcomes in a multiyear study period. Furthermore, projections of total medical costs from a third-party payer perspective may not be very useful beyond a 1-year time horizon due to shifts in drug pricing, reimbursement rates, turnover of plan membership, and changes in benefit design.

**Figure 1:**
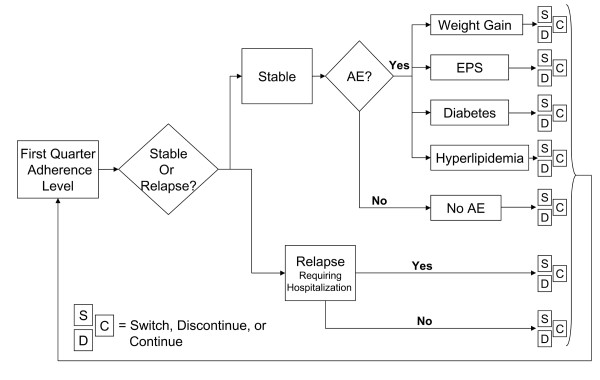
**Conceptual View of MCM Model**.

Figure 1 presents a conceptual overview of the usual treatment for patients living in the community where patients are initiated on specific antipsychotic medications and manifest various adherence levels (fully adherent, partially adherent, or nonadherent). Depending on their adherence level, the patients may (a) remain stable, (b) suffer relapse(s) requiring hospitalization, or (c) relapse(s) not severe enough to warrant psychiatric hospitalization. The patients could potentially experience treatment-emergent adverse events: extrapyramidal symptoms (EPS), clinically significant weight gain (≥ 7%), diabetes, or hyperlipidemia. Depending on benefits and/or adverse events on the initiated medication, the patients and/or their treating physicians decide whether to continue or discontinue the medication. Medication discontinuations involve either a switch to another antipsychotic or discontinuing antipsychotic treatment for awhile. The model takes into account switching patterns, incorporating the primary reason for medication discontinuation (poor efficacy, intolerability, patient decision, or other reasons). As patients with schizophrenia are at a high risk of suicide, the model also incorporates the risk of attempted and completed suicide [60]. The patient's health state at the end of the first quarter constitutes the base for the patient's health state in the next quarter until the end of the fourth quarter (1 year). In addition, certain adverse events (i.e., diabetes and hyperlipidemia) were assumed to remain "with" the patient for the remaining periods, since these adverse events may not disappear within the 1-year timeframe and, therefore, contribute to treatment costs for the remainder of the study period.

### Sequential Bifurcation Test

The MCM model is designed to capture clinically relevant variables for patients with schizophrenia in the usual care setting. However, important clinical variables do not always impact total treatment costs or cost-effectiveness results due to low incidence, low cost, or both. As a result, we used sequential bifurcation [61] to screen all model inputs to determine those variables impacting total treatment costs that warrant focus in sensitivity analyses. Sequential bifurcation is a process that iteratively samples inputs within relevant input ranges and assesses the impact of each input against a predetermined threshold value. For each of the iterations, factors that impact results at or above the threshold value are used in the next iteration. This process continues until there remains no new factor that impacts model outputs by the specified threshold value. Overall, the analyses tested 16 groups with 11 distinct variables examining the impact of variation in over 120 different input assumptions.

The results of the sequential bifurcation tests demonstrated that not all variables that are clinically relevant impact economic outcomes. The suicide rate for patients with schizophrenia is an example of a clinically relevant input, but the sequential bifurcation confirms that it does not impact economic outcomes because of its relatively low incidence rate. In addition, the sequential bifurcation test found that the majority of the costs associated with failed suicide attempts are captured in the treatment cost of an inpatient relapse. Further, cost incurred after a completed suicide are mainly societal and as such, generate no additional costs in our model, and the simulation ends for that patient. Therefore, input assumptions for the suicide rate are modifiable in the MCM model, but this variable is not included in the sensitivity analyses.

### One-Way Sensitivity Analyses

The sequential bifurcation tests indicate that the key economic outcomes of the MCM model include the number/cost of unit health care resources, relapse rates, initial adherence rates, and conditional probabilities of relapse given a history of relapse. As a result, we conducted single variable sensitivity analyses to examine the impact of discrete changes in the value of these variables on the model's results. Specifically, we performed the following 5 analyses:

1. Sensitivity on adherence rates;

2. Sensitivity on adverse event rates;

3. Sensitivity on relapse rates expressed as inpatient hospitalization risk ratios;

4. Sensitivity for olanzapine versus risperidone, changing CATIE relapse risk ratio to achieve desired ICER result.

5. Variation in the cost per day of therapy for generic risperidone.

It should be noted that 1-way sensitivity analysis was not conducted on key input variables that did not vary between the 5 antipsychotic medications, such as the cost of most health care resources.

### Probabilistic Sensitivity Analyses

We conducted 2 multivariable PSAs to examine the uncertainty in the model and the stability of the results. The first PSA allowed the input values for adherence rates, relapse rates, treatment discontinuation rates, and the generic cost of risperidone to be randomly drawn from independent distributions of possible input values. With the exception of the generic cost of risperidone, the range of possible input values was created by setting the minima and maxima of the range to be 50% and +50% of the base case value. The second PSA extended the first analysis by adding distributions around the number and cost of resources consumed for stable patients (no relapse), patients experiencing inpatient relapse, and patients experiencing outpatient relapses. In both PSAs, the results were based on 1,000 cohorts of 1,000 patients each.

### Key Clinical and Economic Input Values

The sequential bifurcation analysis identified a number of key clinical and economic inputs. The remainder of this section reviews the development of the baseline assumption for these key inputs, which were based, when possible, on evidence reported in peer-reviewed articles. Information reported in these articles is used to derive baseline assumptions for each of the 5 antipsychotic medications.

### Adherence Levels

Adherence to antipsychotic therapy in the MCM model is based on the annual medication possession ratio (MPR), the number of days with the medication prescribed by the total number of days in a given period [16,28,30-32]. The MCM model allowed for patients to be categorized into 1 of 3 adherence levels: fully adherent (MPR >/= 80%), partially adherent (60% </= MPR < 80%), or nonadherent (MPR < 60%) [22]. The baseline assumptions of the proportion of patients who fall into the full, partial, or nonadherent categories are based on the information contained in the only published latent class analysis reporting adherence rates of an antipsychotic medication for patients in the United States [62]. In order to derive differential adherence distributions (for fully, partially, or nonadherent patients) for the 5 antipsychotic medications, we made the following assumptions: 1) the results for haloperidol, a typical antipsychotic reported in Ahn [62], represent the lower bound of adherences for the MCM model because the findings are based on Medicaid patients; 2) we then used the annual MPR ratios reported in Ascher-Svanum [31] by medication (olanzapine = 75%; risperidone = 69%; quetiapine = 61%, and haloperidol = 49%) to produce an adjustment factor for each adherence level for these medications; 3) proportion of patients at each adherence level for ziprasidone and aripiprazole were assumed to be equal to quetiapine as in a previous cost-effectiveness study [18]. Table 1, Part A, reports the MCM model's baseline adherence rates by adherence category for each study medication.

**Table 1:** Adherence Input Values

**Part A: Adherence Rates by Medication**				
**Medications**	**Full**	**Partial**	**Non**	

Olanzapine	23%	43%	34%	
Risperidone	21%	39%	40%	Ahn et al., 2007 [62]; Ascher-Svanum et al., 2009 [22]
Quetiapine	19%	35%	46%	
Ziprasidone	19%	35%	46%	Assumed equal to quetiapine
Aripiprazole	19%	35%	46%	Assumed equal to quetiapine

**Part B: Adherence Rate by Level in Cycle Following Relapse**				

**Adherence Level Prior to Relapse**	**Full Adherence After Relapse**	**Partial Adherence After Relapse**	**Non-Adherence After Relapse**	

Full adherence	92.03%	1.45%	6.52%	
Partial adherence	75.00%	12.50%	12.50%	Ascher-Svanum et al., 2009 [22]
Nonadherence	38.70%	9.70%	51.60%	

The MCM model also requires a set of assumptions concerning expected level of adherence in subsequent cycles following a relapse in the previous quarterly cycle. Because of the lack of published data by reporting this information for the study medications, all patients in the MCM model were assumed to change their level of adherence primarily through relapse. Table 1, Part B, reports these baseline assumptions concerning adherence rates in the cycle following a relapse. The variation in baseline assumptions based on the adherence category in previous quarterly cycles were based on a new analysis of the U.S. Schizophrenia Care and Assessment Program (US-SCAP) data conducted to examine how adherence levels change from pre- to post-relapse [22]. US-SCAP is a large, 3-year, prospective, naturalistic, observational, noninterventional, multisite study of persons treated for schizophrenia across the United States [12,63,64].

### Relapse Rates

The MCM model requires a series of assumptions concerning patients' adherence levels and relapse rate for each of the study medications. One study – sponsored by the National Institute of Mental Health (NIMH) – the Clinical Antipsychotic Trials of Intervention Effectiveness (CATIE) [23] provided the data on relapse rates. This large, randomized, double-blind study provides relapse rates for 4 of the 5 antipsychotics included in our model for an 18-month study period. This independent study is the only one to provide relapse data for each of the studied 4 atypical antipsychotics in the treatment of chronically ill schizophrenia patients in the United States. Results from the primary phase of CATIE, phase 1 [23], found significant differences among the antipsychotics for relapses requiring hospitalization, with olanzapine therapy having the lowest risk of relapse (number of hospitalizations/total person-year of exposure). The reported hospitalization risk ratios for the 4 medications of interest were 0.29× for olanzapine, 0.45× for risperidone, 0.66× for quetiapine, and 0.57× for ziprasidone. Table 2, Part A, presents the MCM model's baseline assumptions for the risk of an initial relapse resulting in an inpatient hospitalization by adherence category for each medication. We used the following 3-step process to estimate these relapse rates. First, a baseline relapse rate by adherence level was adopted from a study by Gilmer and colleagues [16] among Medicaid patients. Second, the relapse rates for olanzapine, quetiapine, risperidone, and ziprasidone were derived using the hospitalization risk ratios reported from CATIE phase 1 [23]. Consistent with a prior model comparing the cost-effectiveness of antipsychotics in the treatment of schizophrenia [18], we also assumed that the rates of relapse for aripiprazole are equivalent to ziprasidone. This was done because no comparative data are available for aripiprazole versus the other 4 studied atypicals on relapse rates as the CATIE study did not include aripiprazole. Finally, we assumed a constant proportion of inpatient-to-outpatient rates of relapse by adherence level; 1.0 for fully adherent; 1.13 for partially adherent; and 1.11 for nonadherent for all antipsychotic medications studied [18].

**Table 2:** Relapse Input Values

**Parameter**	**Value**			**Data Source**
**Part A:**				
**Relapse Rates Requiring Hospitalization – For Initial Relapse**	**Full Adherence**	**Partial Adherence**	**Non-Adherence**	

Olanzapine	2.0%	3.6%	5.2%	
Risperidone	3.2%	5.8%	8.8%	Lieberman et al, 2005 [23];
Quetiapine	4.9%	8.8%	14.0%	Gilmer et al, 2004 [16]
Ziprasidone	4.2%	7.4%	11.6%	
Aripiprazole	4.2%	7.4%	11.6%	Assumed equal to ziprasidone

**Relapse Rates Not Requiring Hospitalization**	**Full Adherence**	**Partial Adherence**	**Non-Adherence**	

Olanzapine	2.0%	3.2%	4.8%	Lieberman et al, 2005 [23];
Risperidone	3.2%	5.1%	7.9%	Gilmer et al, 2004 [16];
Quetiapine	4.9%	7.8%	12.6%	Edwards et al, 2005 [18]
Ziprasidone	4.2%	6.6%	10.5%	
Aripiprazole	4.2%	6.6%	10.5%	Assumed equal to ziprasidone

**Part B:**				
**Adjusted Relapse Rates Given a History of Relapse**	**Full Adherence**	**Partial Adherence**	**Non-Adherence**	

Probability given history of 1 relapse	19%	40%	58%	
Probability given history of 2 relapses	36%	75%	100%	Olfson et al., 2000 [65]; Tiihonen et al., 2006 [66]
Probability given history of 3 relapses	42%	88%	100%	

**Part C:**				
**Probability of Suicide Event Given Adherence Level**	**Fully Adherent**	**Partially Adherent**	**Non-Adherent**	

Probability of suicide attempt	0.25%	0.76%	1.00%	Ahn et al., 2007 [62]
Probability suicide attempt is fatal	10.00%			Siris 2001 [60]
Cost of non-fatal suicide attempt	$140 (in addition to relapse costs)			Assumption
Cost of fatal suicide attempt	$0			Assumption

In addition, the MCM model requires a set of conditional probabilities to allow for: 1) multiple outpatient relapses within a single quarter, 2) multiple inpatient relapses within a single quarter, and 3) higher rates of inpatient relapse given a history of inpatient relapse. First, we assumed if a patient had an inpatient relapse, there was a 20% probability of the occurrence of another inpatient relapse during the same quarter [18]. If the first event was an outpatient relapse, then there was a 75% chance of another outpatient relapse during that quarter [18]. Second, the probabilities of having an inpatient relapse given 1 inpatient relapse in a previous quarter across adherence categories was adjusted to reflect the impact of adherence on relapse found in prior research [65,66] which reported that in the 3 months following a relapse, 19% of fully adherent (> 80% MPR) and 43% of nonadherent patients (< 80% MPR) experienced relapses. We set the probability of a second relapse at 19% for patients fully adherent and distributed the probability of a second relapse (43%) between the partially adherent and nonadherent groups weighted by the mean baseline proportion of individuals in each group. These steps result in the baseline assumptions reported in Table 2, Part B. It should be noted that using these baseline rates in the MCM model results in a weighted average number of relapses that is nearly identical to the crude rate of relapse for individuals with a history of 1 relapse reported in the literature (0.47 vs. 0.46) [36].

### Treatment-emergent Adverse Events

The MCM model requires assumptions about the likelihood of patients experiencing 4 types of potential treatment-emergent adverse events: EPS, clinically significant weight gain (≥ 7% weight gain from baseline weight), diabetes, and hyperlipidemia for each medication. Table 3 reports all baseline assumptions concerning adverse events by medication. EPS rates for olanzapine and risperidone are based on results from an integrated analysis of 23 clinical trials that compared incidences of EPS, dystonic, parkinsonian, and akathisia events [67]. EPS rates for quetiapine and ziprasidone are based on package insert information, while the rate for aripiprazole is based on a 1-year randomized, double-blind study comparing olanzapine and aripiprazole in the treatment of patients with schizophrenia [68]. Baseline assumptions concerning potentially clinically significant weight gain for all treatments except aripiprazole are based on the CATIE phase 1 results [23]. Baseline assumptions for event rates for emergent diabetes for olanzapine, risperidone, and quetiapine are based on Lambert et al. [69]. Due to the lack of data for treatment-emergent diabetes for ziprasidone and aripiprazole, we make the assumption that their rates are the lowest rates reported in the Lambert et al. study [69] (equal to typical antipsychotics). The rates for treatment-emergent hyperlipidemia were based on baseline rates reported for all CATIE participants [23] adjusted to rates reported in 2 California Medicaid studies [70,71]. The differential in baseline rates for EPS and potentially clinically significant weight gain for aripiprazole were based upon results of a double-blind, randomized comparative study of aripiprazole versus olanzapine [68]. Finally, the MCM model requires a baseline assumption concerning the proportion of patients developing coronary heart disease (CHD) overall and conditional on having diabetes or metabolic syndrome. The MCM model used a quarterly baseline rate of 0.25% for the probability of developing CHD, calculated to be consistent with the model's 1-year timeframe using the Framingham risk equation [23,72,73] and assumed a relative risk of 2.67 of CHD given diabetes [74] and 4.47 relative risk of CHD given metabolic syndrome [74].

**Table 3:** Adverse Event Values

**Parameter**	**Value**	**Data Source**
**Adverse Event Rates for EPS**		
Olanzapine	15.5%	Carlson et al., 2003 [67]
Risperidone	24.7%	
Quetiapine	8.0%	Package insert, revised 10/2007
Ziprasidone	14.0%	Package insert, revised 07/2007
Aripiprazole	21.0%	Fleischhacker et al., 2008 [68]

**Adverse Event Rates for Clinically Significant Weight Gain (≥ 7%)**		

Olanzapine	30.0%	
Risperidone	14.0%	Lieberman et al., 2005 [23]
Quetiapine	16.0%	
Ziprasidone	7.0%	
Aripiprazole	7.3%	Fleischhacker et al., 2008 [68]

**Adverse Event Rates for Diabetes**		

Olanzapine	3.3%	
Risperidone	3.2%	Lambert et al., 2006 [69]
Quetiapine	3.6%	
Ziprasidone	2.0%	Assumed equal to Lambert et al., 2006 [69] lowest reported rate, that for typicals
Aripiprazole	2.0%	

**Adverse Event Rates for Hyperlipidemia**		

Olanzapine	16.8%	
Risperidone	14.0%	Lieberman et al., 2005 [23]
Quetiapine	14.1%	Lambert et al., 2005 [70]
Ziprasidone	8.1%	Olfson et al., 2006 [71]
Aripiprazole	3.6%	

### Medication Discontinuation Rates

The MCM model allows patients to discontinue therapy for various reasons and from any health state, including stable patients without a treatment-emergent adverse event. The model allows for 4 major reasons for discontinuation: 1) Lack of efficacy, 2) Medication intolerability, 3) Patient decision, and 4) Other reason. Baseline assumptions concerning discontinuation rates from all health states in the model were calculated to yield the annual discontinuation rates based on the survival curves from the 18-month long CATIE phase 1 [23]. The integration of the CATIE phase 1 results and the model states was accomplished by repeated calibration of a multivariable system of equations. The final effect was that the sum of model-specific estimates of discontinuation from all states in the model, including each type of adverse event, matches the annual CATIE phase 1 discontinuation rates for any cause. These annual rates for each study medication are reported in Table 4. The annual discontinuation rate for aripiprazole is based upon a head-to-head trial with olanzapine [68] and the distribution by reason for discontinuation for aripiprazole was created using the same proportions as ziprasidone in CATIE, assuming that ziprasidone and aripiprazole possess similar efficacy and tolerability profiles [18]. Table 4 also reports how the baseline discontinuation rates for each medication are distributed across the 4 reasons for discontinuation [23]. For each medication, the sum of the discontinuation rates across the 4 reasons equals the annual all-cause discontinuation rate.

**Table 4:** Treatment Discontinuation Rates

**Parameter**	**Value**				**Data Source**
**Annual All-Cause Discontinuation Rates**					
Olanzapine	54.0%				
Risperidone	63.0%				Lieberman et al., 2005 [23]
Quetiapine	76.0%				
Ziprasidone	74.0%				
Aripiprazole	61.0%				Fleischhacker et al., 2008 [68]

**Annual Discontinuation Rates by Reason**					

	**Lack of Efficacy**	**Intolerability**	**Patient Decision**	**Other**	

Olanzapine	13%	16%	20%	5%	
Risperidone	22%	10%	22%	9%	Lieberman et al., 2005 [23]
Quetiapine	27%	14%	29%	6%	
Ziprasidone	25%	13%	30%	6%	
Aripiprazole	15%	18%	23%	5%	Fleischhacker et al., 2008 [68]

### Medication Switching Patterns

The MCM model requires a set of assumptions regarding the switching patterns that takes into account the reason for the switch and attempts to choose subsequent treatments that relate to that reason. For example, discontinuation due to EPS would result in a switch to treatments with a more favorable EPS profile. The same approach was used to estimate switching patterns for clinically significant weight gain, diabetes, hyperlipidemia, lack of medication efficacy (a relapse), or patient decision. As such, the options for treatments to "switch to" are dependent on the treatment a patient is "switched from" and are consistent with the comparative efficacy and tolerability of the antipsychotics studied and reported for the CATIE [23-25] and other research [19,75]. Table 5 presents the medication-switch patterns (the medication one is switched from and the medication one is switched to) for each of the 5 reasons for the switching.

**Table 5:** Treatment Switch Patterns by Reason for Switching and by Antipsychotic:

**Medication Switch To →**	**Olanzapine**	**Risperidone**	**Quetiapine**	**Ziprasidone**	**Aripiprazole**	**Clozapine**
**Medication Switched From ↓ by Reason**						
**Lack of Efficacy**						
Olanzapine	0%	20%	10%	20%	20%	30%
Risperidone	30%	0%	20%	20%	20%	10%
Quetiapine	20%	20%	0%	20%	20%	20%
Ziprasidone	30%	20%	20%	0%	30%	0%
Aripiprazole	20%	20%	20%	30%	0%	0%
Clozapine	0%	0%	0%	0%	0%	0%
**Weight Gain**						
Olanzapine	0%	10%	10%	35%	45%	0%
Risperidone	0%	0%	10%	45%	45%	0%
Quetiapine	0%	30%	0%	35%	35%	0%
Ziprasidone	0%	0%	100%	0%	0%	0%
Aripiprazole	0%	0%	0%	5%	95%	0%
Clozapine	0%	20%	0%	40%	40%	0%
**Diabetes**						
Olanzapine	0%	10%	10%	35%	45%	0%
Risperidone	0%	0%	10%	45%	45%	0%
Quetiapine	0%	30%	0%	35%	35%	0%
Ziprasidone	0%	0%	100%	0%	0%	0%
Aripiprazole	0%	0%	0%	5%	95%	0%
Clozapine	0%	20%	0%	40%	40%	0%
**EPS**						
Olanzapine	0%	0%	30%	0%	30%	40%
Risperidone	40%	0%	30%	0%	30%	0%
Quetiapine	50%	0%	0%	0%	40%	10%
Ziprasidone	50%	0%	30%	0%	20%	0%
Aripiprazole	50%	0%	40%	0%	0%	10%
Clozapine	0%	0%	0%	0%	0%	100%
**Hyperlipidemia**						
Olanzapine	0%	10%	10%	35%	45%	0%
Risperidone	0%	0%	10%	45%	45%	0%
Quetiapine	0%	30%	0%	35%	35%	0%
Ziprasidone	0%	0%	100%	0%	0%	0%
Aripiprazole	0%	0%	0%	5%	95%	0%
Clozapine	0%	20%	0%	40%	40%	0%
**Patient Preference**						
Olanzapine	0%	50%	10%	20%	20%	0%
Risperidone	30%	0%	20%	20%	20%	0%
Quetiapine	20%	50%	0%	10%	10%	0%
Ziprasidone	20%	50%	10%	0%	10%	0%
Aripiprazole	20%	50%	10%	10%	0%	0%
Clozapine	0%	0%	0%	0%	0%	100%

### Utility and quality-adjusted life year

Disease-specific utility values for 8 schizophrenia disease states have been reported by Lenert and colleagues [76] using the Positive and Negative Syndrome Scale. Table 6 reports the baseline utility values assigned to each of the 9 possible combinations of adherence levels (full, partial, or nonadherence) and the relapse results (stable, outpatient relapse, or inpatient relapse) required by the MCM model. A panel of 12 independent schizophrenia experts was used to develop these values as follows. First, we surveyed (via email) the panel of experts to determine which of Lenert and colleagues' 8 possible health states best matched the utility of a schizophrenia patient in each of the MCM model's 9 possible adherence/relapse outcomes. Next, we rounded the averaged survey response to the nearest whole number and assigned this number the appropriate utility value reported by Lenert and colleagues [76]. Table 6 also reports baseline assumptions concerning disutility among patients experiencing 1 of the model's 4 treatment-emergent adverse events: EPS, clinically significant weight gain, diabetes, and hyperlipidemia. The disutility multipliers reported for EPS and clinically significant weight gain were derived from those reported by Lenert and colleagues [76]. We assumed that utilities among patients experiencing diabetes or hyperlipidemia were equal to that of patients experiencing EPS, as we are unaware of any peer-reviewed utility information for patients with schizophrenia experiencing diabetes or hyperlipidemia.

**Table 6:** Utility Values for Health States and Disutility Multipliers for Treatment-emergent Adverse Events

**Parameter**	**Value**			**Data Source**
**Health States**	**Full Adherence**	**Partial Adherence**	**Non-Adherence**	

While Stable	0.88	0.75	0.75	Lenert et al., 2004 [76];
Outpatient Relapse	0.74	0.65	0.65	Expert opinion
Inpatient Psychiatric Relapse	0.53	0.53	0.42	
**Treatment-Emergent Adverse Events**				
EPS	0.888			Lenert et al., 2004 [76]
Clinically Significant Weight Gain	0.959			
Diabetes	0.888			Assumption: diabetes, hyperlipidemia, and Metabolic syndrome;
Hyperlipidemia	0.888			utilities equal EPS utility in Lenert et al., 2004 [76]

EPS = extra-pyramidal symptoms

### Medication Costs

The cost of atypical antipsychotic medication is related to daily dose levels, which in turn are linked to patients' illness severity. In order to use comparable medication doses for the treatment of patients with schizophrenia who manifest similar illness severity profiles, we used daily dose levels reported in published, randomized, controlled, schizophrenia studies [23,77,78]. Table 7, Part A, reports baseline model assumptions concerning dosing and cost for each medication. With the exception of generic risperidone, medication costs reflect 2007 net wholesale price (NWP) [79]. We used NWP instead of average wholesale price (AWP) because most third-party payers negotiate price discounts. In addition, we conducted a separate PSA that allowed medication costs to range from 20% above AWP to 50% below AWP for each study medication. These results are not reported because they did not materially change key cost-effectiveness results. Since the cost of generic risperidone is fluctuating at present, we estimated its average cost during the first year post-patent expiry to be at a 58% discount from its 2007 NWP [19].

**Table 7:** Economic Input Parameters

**A: Medication Costs**						
	**Cost**		**Mean Modal Daily Dose (mg)**			

Olanzapine	$15.44		15			NWP Prices, Analysource Data, January 30, 2007 [79]
Risperidone-generic	$5.00		4			Doses: Conley and Mahmoud, 2001 [77];
Quetiapine	$14.79		500			Tunis et al., 2006 [49];
Ziprasidone	$9.81		100			Lieberman et al., 2005 [23]; Kern et al., 2006 [78];
Aripiprazole	$10.92		15			Generic risperidone NWP price = $5.00 per 4 mg/day

**B: Health Service Resource Utilization**						

**Health Service**	Per Stable Quarter*	Per Outpatient Relapse Event*	Per Inpatient Relapse Event*	Extrapyramidal Symptoms (EPS)*	Clinically Significant Weight Gain*	

Hospitalization days	0.0	0.0	11.7**	0.0	0.0	
Day hospital treatment, day	0.0	1.25	1.25	0.0	0.0	
Emergency room visits	0.0	1.0	1.0	0.0	0.0	
Physician visits	3.0	1.0	1.0	1.0	0.5	*Edwards et al., 2005 [18];
Mental health clinic visits	4.5	2.0	2.0	1.0	2.5	**AHRQ HCUP [82]
Home care hour	0.0	2.75	2.75	0.0	0.0	
Group intervention hour	1.5	1.5	1.5	0.0	5.0	
Nutritionist visits	0.0	0.0	0.0	0.0	2.5	

**C: Unit Costs of Health Service Resources**						

Inpatient hospital, per day	$828					AHRQ HUCP [82]
Day hospital treatment, per day	$501					Edwards et al., 2005 [18]
Emergency room visit	$480					
**Outpatient Care**						
Physician visit	$74					
Mental health clinic visit	$75					
Home health care (per hour)	$82					
Group therapy (per hour)	$71					
Nutritionist visit (per hour)	$111					

### Resource Utilization

The model requires resource utilization assumptions for 8 different types of health care services (hospitalization days, day hospital treatment days, emergency room visits, physician visits, mental health clinic visits, home care hours, group intervention hours, and nutritionist visits) across 5 patient outcomes (units per stable quarter, inpatient relapse event, outpatient relapse event, EPS, and potentially clinically significant weight gain). It is assumed that treatment-emergent diabetes and hyperlipidemia would be treated in the normal course of quarterly medical care. As such, there are no discrete units of utilization assigned to these events, but they are represented by aggregated quarterly costs for routine care and additional pharmacy costs [80,81]. Table 7, Part B, reports baseline assumptions for health care utilization in treating 5 patient outcomes: stable quarters (no relapse), per outpatient relapse, per inpatient relapse, EPS, and clinically significant weight gain. The MCM model set baseline length of stay for psychiatric inpatient hospitalization on values reported by the Healthcare Cost and Utilization Project (HCUP) Nationwide Inpatient Sample [82]. All other baseline utilization assumptions are consistent levels reported in prior U.S. cost-effectiveness research [18].

### Health Service Resource Costs

The model requires resource cost assumptions for 3 types of acute health care services (inpatient hospitalization per day, day hospital treatment per day, and emergency room visit) and 5 outpatient health care services (physician visits, mental health clinic visits, home care hours, group intervention hours, and nutritionist visits). These baseline cost assumptions are reported in Table 7, Part C. All unit costs assumptions are inflated to reflect the value of 2007 U.S. dollars using the medical services component of the consumer price index [83].

### Cost of Adverse Events

The MCM model also captures the direct health care cost associated with treating 3 types of treatment-emergent adverse events: diabetes, hyperlipidemia, and EPS. The MCM model assumes that the quarterly cost of all health care utilization associated with the treatment of emergent diabetes is $600 per quarter based on the findings of Leslie and Rosenheck [84]. The baseline assumption for the quarterly costs of statins for hyperlipidemia therapy is $225 and is based on a 50% market share of 40 mg generic statins and a 50% market share of branded statins [80]. The baseline cost of treating EPS with anticholinergics is assumed to be $12 per quarter based on the cost of benztropine (2 mg/day) [18]. Finally, the MCM model assumes all patients, regardless of initiated antipsychotic, undergo metabolic monitoring per published expert consensus guidelines [81] and include lab costs for fasting glucose level or hemoglobin A1c at the time of initiation, 4 months after starting and at 12 months. However, patients with potentially clinically significant weight gain are assumed to incur the cost of undergoing metabolic monitoring every 4 months.

## Model Outcome Measures

### Clinical Outcomes

The MCM model provides estimates of 3 key clinical outcomes: the proportion of patients experiencing outpatient relapse, those experiencing inpatient relapse, and those without an inpatient or outpatient relapse (stable or no relapse). The model also yields estimates of the mean QALYs per patient by medication.

### Economic Outcomes

The main economic outcome of the MCM model is total annual direct health care costs. The model also reports mean total direct health care costs for 4 selected outcomes: cost of stable days, cost of inpatient relapse, cost of outpatient relapse, and cost of adverse events. Finally, the MCM model reports the total annual medication cost of each antipsychotic medication.

### Cost-Effectiveness Information

The cost-effectiveness measure in the model is the incremental cost-effectiveness ratio (ICER), which was calculated as the difference in costs between 2 comparators divided by their difference in QALYs. In this study, we assumed that any ICER in the $50,000–$100,000 range was cost-effective [85].

ICERs have traditionally been calculated relative to no treatment. However, "no treatment" is not an appropriate option for persons with schizophrenia. As a result, the model calculates the ICER for every nondominated treatment.

## Results

Two sets of results are presented in this paper. First, we report the MCM model results using all baseline assumptions. Then we summarize the results of the 5 sensitivity analyses conducted.

### Clinical Outcomes

**Figure 2:**
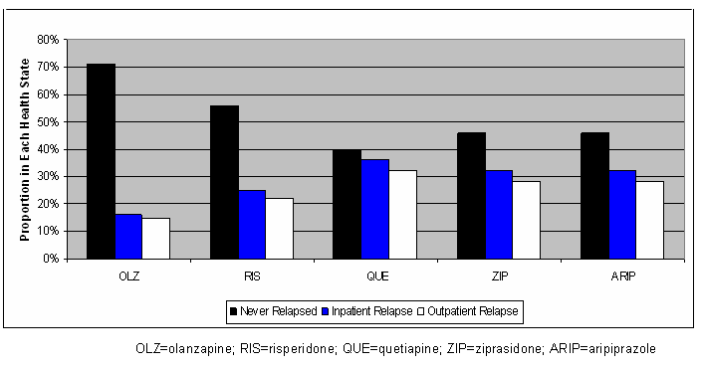
**Base Case Clinical Outcomes**.

**Figure 3:**
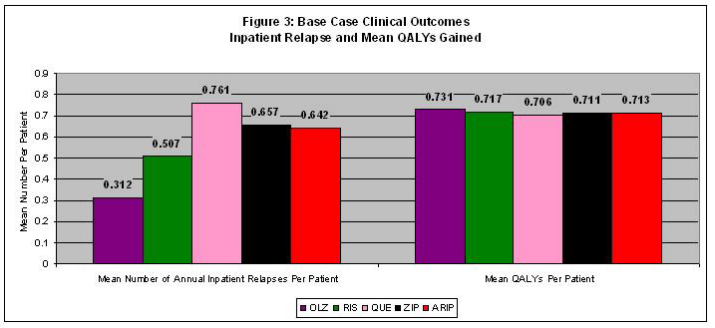
**Base Case Clinical Outcomes – Inpatient Relapse and Mean QALYs Gained**.

Figures 2 and Figure 3 display a complete set of base case clinical results for the 5 comparators in the model. For the base case, Figure 2 indicates that olanzapine would result in more patients never experiencing relapse, fewer inpatient relapses, and fewer outpatient relapses. In addition, Figure 3 indicates that olanzapine would result in a lower number of relapses per patient and the highest QALY of any medication studied.

### Economic Outcomes

**Figure 4:**
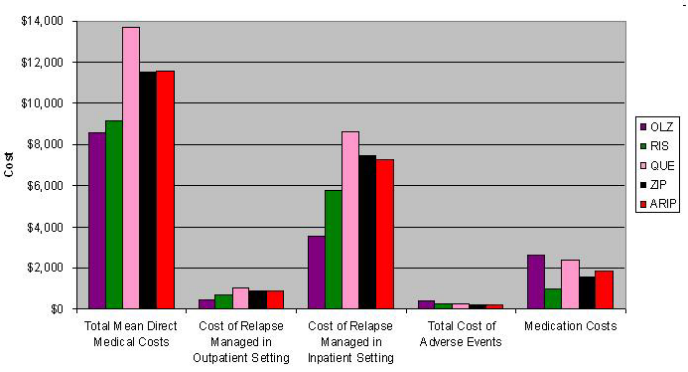
**Base Case Economic Outcomes**.

Figure 4 displays base case estimates of the total mean direct medical cost, as well as predicted costs for each of the key cost components for all comparators in the model. For total mean direct medical cost, the base case of the model predicted that olanzapine results in the least costly option among the 5 comparators. Figure 4 also demonstrates the mean annual direct costs varied by selected cost component by medication. For example, olanzapine had the highest annual medication acquisition cost but the lowest annual mean cost of treating relapse of the 5 comparators.

### Cost-Effectiveness

The incremental cost per QALY gained of 1 comparator versus another is a widely recognized metric of cost effectiveness. A treatment that produces more QALY at a higher cost may be cost effective if the resulting ICER is below a stated threshold. As seen in Figure 3 and Figure 4, olanzapine was predicted to result in the highest mean QALY per patient and the lowest total mean direct medical cost. As a result, from a cost-effectiveness perspective, olanzapine was the dominant therapy in terms of cost/QALY gained because it is predicted to produce more QALYs at a lower cost. Moreover, Figures 3 and 4 show that olanzapine was dominant in 1-to-1 comparisons with each comparator in the same manner. Finally, risperidone dominated quetiapine, ziprasidone, and aripiprazole producing more QALY at a lower cost. One-way and probabilistic sensitivity analyses were conducted to examine the stability of the base case results.

### One-Way Sensitivity Analyses Results

One-way sensitivity analysis was performed to assess the impact of important variables on model outcomes and patient subgroups. First, Table 8, Test 1, reports the results of the sensitivity analysis for adherence subgroups simulated in the model. These results were calculated by assuming that all patients had the same adherence level. First, we simulated the model with a cohort of fully adherent patients and recorded the mean total direct medical cost by medication and the cost per QALY for olanzapine. The same process was applied to a cohort of partially adherent patients and then a cohort of nonadherent patients. The results from this sensitivity test indicate that risperidone has the lowest total cost if all patients remain fully adherent ($7,932) or partially adherent ($9,365), but that olanzapine has the lowest total cost if all patients are nonadherent ($6,699). In addition, the results for cost per QALY indicate that olanzapine is the dominant choice for nonadherent patients and a cost-effective option for partially adherent patients (ICER of $44,718) and for fully adherent patients (ICER of $82,519).

**Table 8:** Test 1: Sensitivity – Adherence Subgroups

	Base Case Mean Cost (ICER)	Fully Adherent Patients Total Mean Cost (ICER)	Partially Adherent Patients Total Mean Cost (ICER)	Nonadherent Total Mean Cost (ICER)
OLZ	$8544 (Dominant)	$9349 ($87,323)	$9723 ($52,277)	$6699 (Dominant)
RIS	$9080	$7877	$9290 (Base)	$9367
QUE	$13619	$11856	$13473	$14267
ZIP	$11414	$9882	$11301	$12003
ARIP	$11603	$10191	$11547	$12090

ICER = incremental cost-effectiveness ratio; OLZ = olanzapine; RIS = risperidone; QUE = quetiapine; ZIP = ziprasidone; ARIP = aripiprazole

Table 9, Test 2 displays results for 1-way analysis was performed to assess the impact of 2 treatment-emergent adverse event rates on model outcomes: diabetes and hyperlipidemia. Literature-based rates [84] for treatment-emergent diabetes were used as upper bounds for all comparators. The upper limits for hyperlipidemia were based upon a California Medicaid Study [71]. The model results indicated that olanzapine remained the dominant strategy (highest mean QALY and lowest mean direct cost) when incorporating alternate adverse event rates that were higher than the base case rates. Sequential bifurcation indicated that EPS and treatment-emergent clinically significant weight gain did not directly impact model results and were not included in the sensitivity analysis.

**Table 9:** Test 2: Adverse Event Rates

	Treatment-Emergent Diabetes				Treatment-Emergent Hyperlipidemia			
	
	New Event Rate* (Base Case Rate)	Total Mean Cost	QALYs	ICER Cost/QALYs	New Event Rate** (Base Case Value)	Total Mean Cost	QALYs	ICER Cost/QALYs
OLZ	4.6% (3.3%)	$8567	0.733	Dominant	21.8% (16.8%)	$8582	0.731	Dominant
RIS	4.1% (3.2%)	$9095	0.719	-	21.4% (14.0%)	$9122	0.717	-
QUE	4.3% (3.6%)	$13628	0.708	-	21.3% (14.1%)	$13666	0.706	-
ZIP	4.1% (2.0%)	$11445	0.715	-	19.6% (8.1%)	$11478	0.711	-
ARIP	4.1% (2.0%)	$11632	0.710	-	16.7% (3.6%)	$11678	0.707	-

*Source: Leslie and Rosenheck, 2005 [84]

**Source: Olfson et al., 2006 [70]

QALYs = quality-adjusted life years; ICER = incremental cost-effectiveness ratio; OLZ = olanzapine; RIS = risperidone; QUE = quetiapine; ZIP = ziprasidone; ARIP = aripiprazole

Further analysis showed that olanzapine remained dominant when the rates of diabetes for aripiprazole and ziprasidone were kept at the base case value and all others were increased.

Table 10, Test 3, reports the results of sensitivity analysis associated with changing the rates of relapse expressed as hospitalization risk ratios. The first column of this table shows the base case hospitalization risk ratio reported in the CATIE, phase 1 [23]. This sensitivity analysis was performed by increasing the hospitalization risk ratio of olanzapine to each comparator which has the effect of increasing the costs of olanzapine versus each comparator. The values in the second column show the hospitalization risk ratios for each comparator required to make the total mean direct cost of olanzapine therapy roughly the same as the comparator. The values in the third column are the hospitalization risk ratios at which olanzapine was more costly, but relatively cost-effective in terms of cost per QALY (approximately $50,000).

**Table 10:** Test 3: Sensitivity Analysis – Changing CATIE Relapse Risk Ratios

	Olanzapine/CATIE Ratio	Cost-Neutral Ratio	Cost-Effective Ratio (ICER @ $50000)
RIS	0.64*	0.65	0.73 ($45,385)
QUE	0.44	0.88	0.89 ($60,767)
ZIP	0.57	0.75	0.80 ($43,236)
ARIP	0.57	0.77	0.80 ($44,187)

*Base case ratio from CATIE Phase 1 [23] which reported hospitalization risk ratios normalized to 1 person year of exposure: RIS = 0.45, OLZ = 0.29 thus olanzapine/risperidone = 0.64.

CATIE = Clinical Antipsychotic Trials of Intervention Effectiveness; ICER = incremental cost-effectiveness ratio; RIS = risperidone; QUE = quetiapine; ZIP = ziprasidone; ARIP = aripiprazole

Table 11, Test 4, reports results of the first of 2 head-to-head analyses between olanzapine and risperidone. The results in Table 11, Test 4, answer the following questions: At what relative rates of inpatient relapse does risperidone become cost-neutral when compared to olanzapine, and at what relative rate of inpatient relapse does risperidone dominate olanzapine? The first row of Table 11, Test 4, reports the base case results for comparison. The second row indicates that the hospitalization risk ratio of risperidone compared to olanzapine would need to drop to 1.54 for the 2 medications to be cost-neutral. The final row indicates that the hospitalization risk ratio of risperidone compared to olanzapine would have to be 0.70 for risperidone to begin dominance of olanzapine.

**Table 11:** Test 4: Sensitivity Analysis Olanzapine Versus Risperidone: Changing CATIE Relapse Risk Ratios to Achieve Desired ICER Result

	ICER	
RIS/OLZ Ratio	OLZ	RIS
1.55 (Base Case)*	Dominant	-
1.54	Cost-Neutral	
0.70	Effectiveness-Neutral	

*Base case ratio from CATIE Phase 1 [23] which reported hospitalization risk ratios normalized to 1 person year of exposure: RIS = 0.45, OLZ = 0.29, and RIS/OLZ = 1.55.

ICER = incremental cost-effectiveness ratio; RIS = risperidone; OLZ = olanzapine

Table 12, Test 5, presents results of the second head-to-head analysis between olanzapine and risperidone and reports the sensitivity analysis associated with changing the generic price of risperidone below baseline rate of $5 per day. The second row of this table assumes that risperidone is reduced to 13¢ per day (initiatives by major U.S. retailers). At this price, olanzapine remains cost-effective ($10,246 per QALY) relative to risperidone. Finally the last row indicates that olanzapine remains cost-effective even if risperidone is acquired at zero cost.

**Table 12:** Test 5: Sensitivity Analysis Olanzapine Versus Risperidone: Changing Only the Cost of Generic Risperidone

Cost Per Day of Therapy for Generic Risperidone	OLZ Mean Annual Direct Cost	RIS Mean Annual Direct Cost	ICER
$5.00 (Base)	$8581	$9120	OLZ Dominant
$0.13	$8557	$8408	$10,246
$0	$8557	$8389	$11,509

OLZ = olanzapine; RIS = risperidone; ICER = incremental cost-effectiveness ratio

#### Probabilistic Sensitivity Analysis Results

**Figure 5:**
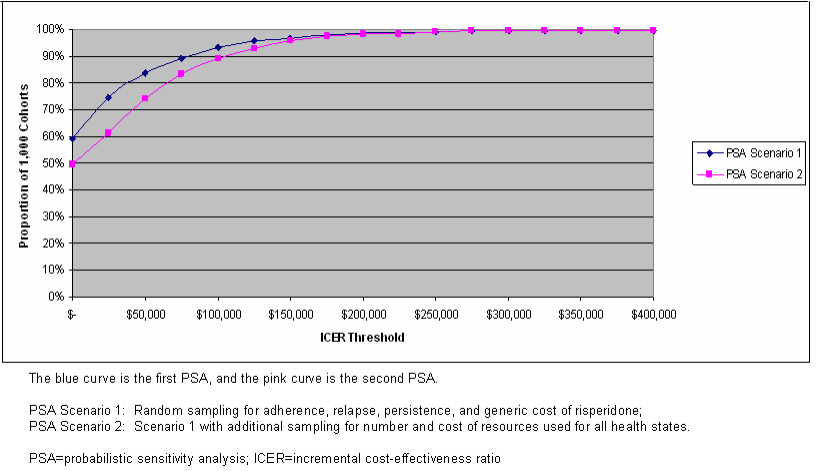
**Proportion of Cohorts At or Below Selected ICER Thresholds**.

Figure 5 reports the percentile of the cohorts, in the head-to-head comparison of olanzapine to risperidone, which had an ICER below the selected cost thresholds in $50,000 increments, starting with all cohorts where olanzapine was found to be dominant graphed at $0. The results of the first PSA (randomly sampled input values for adherence rates, relapse rates, treatment discontinuation rates, and the generic cost of risperidone) are displayed as the lower curve, while the upper curve reports the results of the second PSA (adding randomly sampled input values for the number and cost of resources consumed for stable patients (no relapse), patients experiencing inpatient relapse, and patients experiencing outpatient relapses to the variables in the first PSA). The results of the first PSA (upper curve in Figure 5) indicate that olanzapine compared to risperidone was cost saving (dominant) in 59% of the 1,000 cohorts simulated. Further, the results of the first PSA found that olanzapine compared to risperidone had an ICER of $50,000 or less in 84% of the cohorts simulated, an ICER of $100,000 or less in 93% of the cohorts, and an ICER of $125,000 or less in approximately 96% of the cohorts simulated. The results of the second PSA (lower curve in Figure 5) indicates that even a greater proportion of the 1,000 cohorts met each of the selected ICER thresholds than in the first PSA in the direct comparison of olanzapine to risperidone. In the second PSA, olanzapine was reported to be cost-saving in 50% of the cohorts and had an ICER of $50,000 or less in 74% of the cohorts, while approximately 93% of the cohorts had an ICER of $125,000 or less. Figure 5 reports the proportion of cohorts that selected cost-effectiveness thresholds between $0 and $400,000 because a recent review of cost-effective research in the United States suggested that no single cost-effectiveness threshold is necessarily appropriate and that U.S. policy makers often use different thresholds for different policy issues [85].

## Discussion

This is the first cost-effectiveness analysis to compare oral generic risperidone with various oral atypical antipsychotics in the usual treatment of patients with schizophrenia in the United States. Results of the MCM model's baseline assumptions, along with results of selected sensitivity analyses, found olanzapine to be a cost-effective therapeutic strategy for patients with schizophrenia, even if a 30-day pharmacy fill of oral generic risperidone were to cost $4 per month or less. The MCM model's baseline assumptions predict that the utilization of olanzapine provides better clinical outcomes and lower total health care costs compared to generic risperidone, quetiapine, ziprasidone, and aripiprazole. Furthermore, the latter 3 medications were dominated by both olanzapine and generic risperidone. Specifically, compared to the second most cost-effective treatment strategy (generic risperidone), olanzapine was associated with a lower rate of inpatient relapse (16% vs. 25%), with higher QALY (0.733 vs. 0.719) and with lower total health care cost ($8,544 vs. $9,080).

Sequential bifurcation found that the relapse rate resulting in either inpatient hospitalization or outpatient care is the most significant baseline assumption in the MCM model. Because olanzapine therapy offers patients a lower propensity for relapse than its comparators, a sensitivity analysis was conducted to determine the change in relapse rates required by risperidone to make olanzapine cost-neutral and then just cost-effective. This head-to-head comparison indicated that risperidone is cost-neutral if the hospitalization risk ratio is reduced to 1.54 (RIS/OLZ) from the baseline assumption of 1.55, and risperidone achieves dominance only after the hospitalization risk ratio is reduced to 0.70. This suggests that risperidone would need to generate a 30% risk reduction in hospitalization relapse rates relative to olanzapine. The implication of this sensitivity analysis is that olanzapine is a dominant or cost-effective treatment option over a wide range of relapse rates that likely represent a plausible range of clinical scenarios for patients with schizophrenia.

Additional sensitivity tests indicated that the impact of CHD events, as driven by treatment-emergent diabetes and hyperlipidemia, had minimal impact on overall mean annual medical cost or cost per QALY. For example, the net impact of increasing the rate of treatment-emergent diabetes for olanzapine from the base rate of 3.3% [69] to 4.6%, the value of Leslie and Rosenheck [84], only added $19 annually to the total medical cost for olanzapine. Further, increasing the base rate of hyperlipidemia to a higher, literature-based value increased the mean annual direct medical cost of olanzapine by $38 [71]. These results remain robust despite the fact that they are assumed to occur in the first quarter of treatment initiation, rather than weighing the increase in the rates over the 4 quarterly cycles.

In summary, the cost-effectiveness estimates in our MCM model are sensitive to the baseline assumption that in the treatment of patients with schizophrenia, olanzapine is associated with a lower risk of relapse compared to risperidone, quetiapine, ziprasidone, and aripiprazole. Since the cost of relapse is the core driver, it is important to underscore that the relapse rates in this model were based on the NIMH-sponsored CATIE trial [23]. In addition, this MCM model's baseline assumption is supported by other research, conducted across geographies and methodologies, in which olanzapine was found to achieve lower relapse rates or have longer time to relapse compared to risperidone [64,85-87], quetiapine [40,88-90], and ziprasidone [39,48].

It is also notable that although we used the CATIE findings to support the model's assumption that olanzapine is more effective than the comparators (risperidone, quetiapine, ziprasidone, and aripiprazole), our assumption received recently additional support from the first meta analysis of head-to-head comparisons of 9 second-generation antipsychotics in the treatment of schizophrenia [91]. That comprehensive meta-analysis, which included the 5 antipsychotics studied in our model, also showed that olanzapine proved superior to risperidone, quetiapine, ziprasidone, and aripiprazole. In addition, the authors examined potential sponsorship bias and concluded that exclusion of studies sponsored by pharmaceutical companies did not change the results.

Findings of this cost-effectiveness analysis are consistent with prior research [13,17,18,57,92,93] that also found psychiatric hospitalization to be the largest cost component in the treatment of schizophrenia and medication costs to comprise the second most costly type of resource [17,18]. Also consistent with prior research [19] were the findings that treatment-emergent EPS and diabetes following initiation of therapy with atypical antipsychotics do not lead to substantial cost implications because of their low incidence. Most importantly, current findings are consistent with other cost-effectiveness studies [49,58,92-97] showing that olanzapine therapy is more effective and less or as costly (total direct medical costs) compared to studied atypical antipsychotics, because olanzapine is associated with a lower rate of inpatient hospitalization, the main driver of the cost differentials.

Since our cost-effectiveness model is sponsored by Eli Lilly and Company, the manufacturer of olanzapine, skepticism about potentially biased industry-sponsored economic models prevails [98,99]. However, the findings reported in this paper are consistent with recent cost-effectiveness analysis conducted by Leeuwenkamp and colleagues [93] and sponsored by Organon and Pfizer. That study found that initiation with olanzapine (compared to risperidone) lowered the number of relapses at 1 year and increased the proportion of patients remaining on treatment. As a result, the study concluded that starting with olanzapine rather than risperidone may result in more time without symptoms and lower nonmedication health care costs during the first year of treatment. Another independent cost-effectiveness analysis [92], comparing various antipsychotics, including oral olanzapine, risperidone, quetiapine, and ziprasidone, found olanzapine to be the most effective treatment with the highest proportion of patients remaining free of psychiatric hospitalization. In this study, quetiapine and ziprasidone were found to be dominated by olanzapine while oral risperidone was found to be less effective and less costly but with higher incremental cost-effectiveness ratio than olanzapine.

The results reported in this paper are, however, inconsistent with 4 cost-effectiveness models in the literature. These studies found risperidone was more cost-effective than all other comparators including olanzapine [18,100-102]. These models assumed, however, that olanzapine and risperidone therapy achieved the same medication adherence level [18,100,102] and the same risk of relapse [18,100,101]. In addition, these models assumed that risperidone-treated patients had a shorter length of hospital stay than olanzapine-treated patients and that risperidone is more efficacious than olanzapine [102]. The sensitivity analysis reported in this paper suggest that risperidone would be a cost-effective treatment strategy if we assumed different baseline inputs concerning relapse rates, length of hospital stay, and adherence levels.

The MCM model developed and tested in this paper has a number of strengths. The baseline assumptions are documented and transparent, using inputs and key model outputs that are relevant for comparative economic evaluation of atypical antipsychotics in the treatment of schizophrenia. The structure of the model reflects the essential features of the disease and its treatment processes by simulating the dynamic nature of usual care where patients switch, continue, discontinue, and restart their antipsychotics. The baseline assumptions are relatively conservative, using input parameters that are based on the published literature and evidence available in the public domain. For example, we have chosen a relatively low number of inpatient days of hospitalizations associated with relapses compared to other models (11.7 vs. 23.1) [18,82]. Auxiliary sensitivity analysis showed that increasing the number of inpatient days strengthened olanzapine's cost-effectiveness and dominance. The model allows for changes in all input parameters shown to impact the costs of treating schizophrenia using sequential bifurcation tests. The model findings are also robust as demonstrated by a diverse sensitivity analyses to assess the potential for variation in the model results. And lastly, although our microsimulation model may seem complex, it is important to note that simpler methods implicitly need the same inputs but tend to hide the assumptions that are explicit in our model. We feel that by being transparent we more accurately capture the complex and dynamic nature of the disease and its treatment processes.

Our model has, however, a number of limitations. First, lack of published medical literature for some model input parameters (e.g., QALYs by health states and adherence levels) required using expert panel opinions. In addition, lack of head-to-head randomized studies comparing all 5 studied SGAs required making assumptions that need further study (e.g., that aripiprazole and ziprasidone have the same clinical and safety features). Second, the model does not include all SGAs currently available in the United States, thus excludes clozapine and paliperidone. This exclusion was made a priori since this model focused only on the most frequently used SGAs, and clozapine and paliperidone are used infrequently in the United States. Third, the model used a 1-year time horizon although schizophrenia is a life-long illness. While this follow-up duration is used in most other schizophrenia cost-effectiveness models, it may not be sufficiently long to observe changes in costs and outcomes over the course of a chronic illness. In particular, it may also not allow for accurate assessment of specific treatment-emergent adverse events such as CHD and tardive dyskinesia which usually take longer to develop. Indeed, when the CATIE data was used to estimate the 10-year CHD risk for each of the treatment groups [103], it found this risk has significantly increased for olanzapine-treated patients (+0.5%), whereas it significantly decreased for the ziprasidone and risperidone treatment groups (-0.6%). It is notable, however, that when estimating 10-year CHD risk, one assumes the patients will continue on the current antipsychotic during the following 10 years. This is a questionable assumption considering the highly dynamic and changing nature of treatment for schizophrenia in the United States. It is also important to recognize there is only 1 randomized longitudinal study comparing actual cardiac adverse events in patients treated with different antipsychotics rather than on projected estimates of CHD risk. This was the Ziprasidone Observational Study of Cardiac Outcomes (ZODIAC) [48], a 1-year study of 18,154 patients with schizophrenia, conducted in 18 countries. This study did not find significant differences between the treatment groups with respect to cardiac events (e.g., incidence of cardiovascular mortality or hospitalization for various cardiac conditions). Moreover, consistent with our model's assumptions, ZODIAC found a significantly higher rate of treatment discontinuation and a higher rate of psychiatric hospitalization for the ziprasidone-treated patients compared to the olanzapine treatment group. ZODIAC was, however, only 1 year long, thus not sufficiently long to capture changes in cardiometabolic risk in patients treated with various antipsychotics.

A fourth limitation of the model is its focus on direct cost and exclusion of indirect cost, which can be substantial in the treatment of schizophrenia [3]. Finally, the model did not take into account that some patients have pre-existing adverse events and conditions, including diabetes and hyperlipidemia [104,105], which may impact future costs and outcomes. Additional research is needed to help identify which patients with what profiles respond best to which antipsychotic after failure on specific medications for what reasons.

Results of our cost-effectiveness model also need to be evaluated in a larger context, considering that we focused only on specific SGAs and did not address the comparative cost-effectiveness of first- and second-generation antipsychotics (FGAs and SGAs). This is a topic of much debate about whether the more costly SGAs have superior benefits compared with lower-cost FGAs. This debate may, however, become less relevant for payers, including U.S. payers who may have little incentive to use FGAs following patent expiry of SGAs, such as risperidone and their availability in generic form and lower cost. Nonetheless, studies have reached different conclusions regarding the cost-effectiveness of 1 or more SGAs versus FGAs [17,49,57]. The cost-effectiveness analysis of the CATIE [17] showed that a low potency FGA (perphenazine) was more cost-effective than SGAs, including olanzapine. Another study, conducted in the UK (CUtLASS1) [57], also reported that FGAs may be cost-saving and associated with a gain in QALYs compared with SGAs. This ongoing debate was further fueled by findings of a new comprehensive meta-analysis of SGAs versus FGAs [106] showing that 4 specific SGAs (clozapine, amisulpride, olanzapine, and risperidone) are better than FGAs for overall efficacy, whereas the other SGAs (including quetiapine, ziprasidone, and aripiprazole) are not better. The authors suggested abandoning the classification of antipsychotics into FGA and SGA since each is not a homogeneous class, and improper generalization creates confusion. They pointed out that SGAs differ on many properties, including efficacy, adverse events, cost, and pharmacology. Our cost-effectiveness model avoided comparisons between drug classes and focused instead on 5 specific SGAs that differ in their efficacy, adverse events, pharmacology, and cost.

## Conclusion

The utilization of olanzapine is predicted in this microsimulation model to result in better clinical outcomes and lower mean total health care costs – from the perspective of payers in the U.S. health care system – compared to generic risperidone, quetiapine, ziprasidone, and aripiprazole. Olanzapine is, therefore, projected to be a cost-effective therapeutic option for patients with schizophrenia in the United States, even with oral risperidone available in generic form and cost. This model simulates real-world treatment processes and provides projections that should be used only to inform decision-making processes from the U.S. health care system perspective. Although current findings are consistent with several previous studies, this model – as any other economic model – will require revision and validation of baseline assumptions when new and additional relevant scientific data are available.

## Competing interests

Haya Ascher-Svanum, Anthony Lawson, and Robert Conley are all full-time employees and minor shareholders of Eli Lilly and Company. Nicolas Furiak, Robert Klein, Lee Smolen, and Steven Culler have consulting agreements with Eli Lilly and Company.

## Authors' contributions

NMF developed the model, conducted the sensitivity analyses, interpreted the results, and helped draft the manuscript. HAS helped with model development, interpretation of the results, and preparation of the manuscript. RWK and LJS helped develop the model and its sensitivity analyses. AHL, RRC, and SDC helped interpret the results and assisted with manuscript preparation and revision. All authors read and approved the final manuscript.
